# Identification of biomarkers associated with Parkinson's disease by gene expression profiling studies and bioinformatics analysis

**DOI:** 10.3934/Neuroscience.2019.4.333

**Published:** 2019-12-26

**Authors:** Marios G. Krokidis

**Affiliations:** Bioinformatics and Human Electrophysiology Laboratory, Department of Informatics, Ionian University, Greece

**Keywords:** Parkinson's disease, network analysis, gene expression, bioinformatics, micro-RNAs

## Abstract

Parkinson's disease (PD) is associated with a selective loss of the neurons in the midbrain area called the substantia nigra pars compacta and the loss of projecting nerve fibers in the striatum. Predominant pathological hallmarks of PD are the degeneration of discrete neuronal populations and progressive accumulation of α-synuclein–containing intracytoplasmic inclusions called Lewy bodies and dystrophic Lewy neuritis. There is currently no therapy to terminate or delay the neurodegenerative process as the exact mechanisms underlying the pathogenesis of PD require further investigation. The identification and validation of novel biomarkers for the diagnosis of PD is a great challenge using contemporary approaches and optimizing sampling handling as well as interpretation using bioinformatics analysis. In this review, recent evidences associated with multi-omic data-sets and molecular mechanisms underlying PD are examined. A combined mapping of several transcriptional evidences could establish a patient-specific signature for early diagnose of PD though eligible systems biology tools, which can also help develop effective drug-based therapeutic approaches.

## 1. Introduction

Parkinson's disease (PD) is a progressive age-dependent neurodegenerative disorder that affects an estimated 1% of the population over 50. It is clinically manifested by resting tremor, cogwheel rigidity, bradykinesia, and postural instability, while the motor syndrome of PD is highly associated with the loss of dopaminergic (DAergic) neurons [1]. On the other hand, non-motor symptoms are highly presented, including cognitive deficits, depression, and emotional abnormalities. Intraneuronal inclusions called Lewy bodies characterize the pathology of the disorder and contain abnormal aggregates of the presynaptic protein alpha-synuclein as their main component additionally to ubiquitin and neurofilaments. One of the main factors associated with PD is the aging and deregulation of cellular processes related to highly vulnerability of dopaminergic neurons [2]. Degeneration of various neuronal types is a well-known feature in PD while DAergic neuronal loss in the substantia nigra pars compacta plays an essential role in the characteristic expression of motor symptoms and driven-symptomatic therapies [3]. Oxidative stress is highly involved in the degeneration of dopaminergic neurons in PD. Among ROS-generating enzymes, tyrosine hydroxylase and monoamine oxidase affect neurons and prone to oxidative stress, while in the brains of PD patients increased levels of proinflammatory cytokines have been observed [4]. Metabolism of dopamine itself as well as neuroinflammatory cells and mitochondrial dysfunction are implicated in ROS production [5]. The causes of neuro-inflammation are widely known in the pathology of PD and concurrent clinical results further support the incidence of systemic inflammation and related oxidative stress although molecular pathways for attenuation of the disease have not yet been accomplished [6],[7]. This review presents the availability of high-throughput technologies and bioinformatics dedicated to clinical applications in PD with emphasis on identifying the molecular key factors associated with the pathogenesis of the disease.

## 2. Genetics of Parkinson's disease

**Figure 1.**
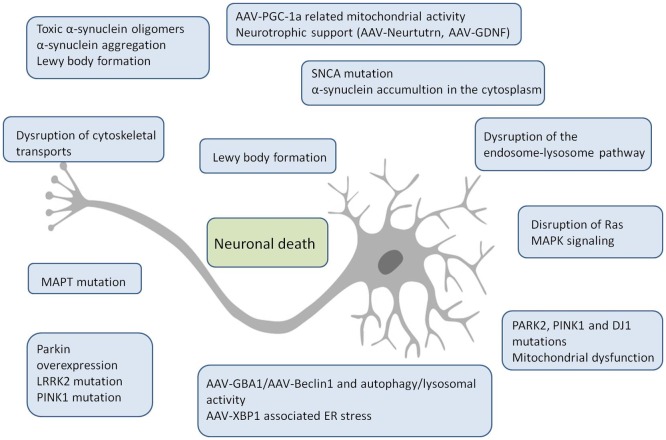
Genetic causes of Parkinsonism and pathways related to Parkinson disease.

Providing an established definition, a biomarker is a measured indicator of normal biological processes or pathogenic condition. Specificity, selectivity, reproducibility and easy-collection are important factors that ultimately determine the diagnostic utility of a biological marker. Focusing on PD diagnosis, the baseline evidence is the type of biomarker requested while ideal molecular markers may be premotor, prodromal or motor stage biomarkers. Appropriate biomarkers could also be classified as clinical, genetic, proteomic or biochemical along with imaging evidences. On the other hand, the identification of applicable biomarkers is fairly difficult since clinical recognition occurs after degeneration of a variety of SN neurons [8]. The majority of our knowledge of the molecular mechanisms of PD has emerged from the identification of key genes involved in the etiology and pathology of the disease (Figure 1). The expansion of the α-synuclein pathology, as well as disturbances of lysosomal and mitochondrial activities, seem to play critical roles in pathogenesis [9],[10].

As it is widely known that upon a significantly increased level of substantia nigra neuronal degeneration the clinical diagnosis of PD can be performed, it is necessary to reveal characteristic diagnostic criteria for the disease as an important basis before clinical symptoms occur. Among these, premotor stage molecular evidences, susceptibility markers and undoubtedly motor stage indicators in an entire diagnostic panel or potential clinical and imaging biomarkers should be included [11]. These incidents should also follow the determination of the efficacy of individual therapies. A comprehensive overview of the expression profiles of particular cells and the topological composition of cell types has the potential to shed light on the differences in brain deterioration and spread of neuropathology observed among PD patients. Genome-wide research may clarify to the mechanism of PD while disagreements about gene regulation and related pathways have been implicated in the etiology of the disease [12]. Even though PD is mainly due to sporadic disorders, a variety of genes have been linked to rare monogenic forms of the disease such as α-synuclein, leucine-rich repeat kinase 2 (LRRK2), parkin, PTEN-induced kinase 1 (PINK1) and DJ-1. Accumulated data show that Parkin (PARK2) and PNK1 are suspected of an autosomal-recessive trait of the disorder [13]–[15].

Exon-deletion mutations in *PINK1*a are associated with Parkinsonism that slowly progresses associated with limited doses of L-DOPA [16]. Similarly, exonic deletion or duplication mutations in *PARK2* (the gene that encodes parkin) are linked with Parkinsonism with daily variations whereas genomic duplications in *SNCA* (the gene that encodes α-synuclein) or prevalent Ala30Pro and Ala53Thr substitutions cause nigral neuronal loss [17],[18]. Sporadic PD has been linked with pathogenic missense mutations on *LRRK2* (Arg1441Cys/His, Ile2012Thr or Gly2019Ser substitution) leading to neurofibrillary tangles and predominant Lewy body abnormalities [19]. Important mutations in *DJ1* and *UCHL1* (ubiquitin carboxyl-terminal esterase L1) associated with either delayed progressive Parkinsonism or sporadic symptoms expression, respectively, should be emphasized here. Leu166Pro substitution related to *DJ1* may lead to Parkinsonism with dementia or amyotrophy [18]. Furthermore, mutations in GCH1 (GTP cyclohydrolase 1) and DNAJC6 have been assessed as risk factors for PD [20],[21].

## 3. Gene expression studies approached for PD biomarkers identification

A plethora of gene expression studied has been conducted to unravel key genes in PD. In a recent study by Jiang et al., microarray datasets of patients with PD from the Gene Expression Omnibus database were downloaded and alterations between PD and normal groups were compared. From 1229 differential expressed genes nine of them were determined that may serve as potential biomarkers of PD, among them *PTGDS, SLC25A20* and *LRRN3* [22]. In order to investigate the key regulators of the PD transcriptional networks in the substantia nigra, a meta-analysis of 8 different studies was carried out including a comparison of 83 postmortem brain samples from patients with PD with 70 normal controls without PD [23]. After applying multiscale gene network analysis to the combined data set, 946 differentially expressed genes were observed that had never been previously associated with the disease. These new evidences are involved in synaptic function, spinal cord development, dopamine metabolism and embryonic digit morphogenesis while STMN2 was identified as a significant factor contributing to synaptic trafficking and the regulation of α-synuclein modification cascade [23].

Associations between PD and alterations in pathways that regulate lipid metabolism and mitochondrial dysregulation have been identified in an outstanding work by Chi et al. Upon meta-analysis of multiple datasets obtained from the Gene Expression Omnibus database, including substantia nigra and peripheral blood samples, the levels of MAPK8, CDC42, NDUFS1, COX4I1, and SDHC were found to be downregulated [24]. Six candidate genes, SNCA, COX17, COX4I1, COX7B, COX6A1 and ATP5J, were selected from 276 differentially expressed transcripts in association with ATP production and oxidative phoshorylation regulation by using Metascape, GO enrichment analysis and KEGG pathway enrichment analysis [25]. DNA methylation data and gene expression data were utilized by 205 PD patients and 233 healthy controls to identify connections between transcriptomic alterations and epigenetic modification in PD as a potential biological blood marker, and unexpectedly, eighty-five significantly hypo-methylated and upregulated molecules were determined in PD patients [26]. eEF1A1, CASK and PSMD6 that are linked to PARK2 activity in the cell were identified as important genes with an increased function role in PD after analysis of microarray data from samples of induced pluripotent stem cells (iPSCs) derived from PD patients and mature neuronal cells differentiated from these iPSCs [27].

Multiple miRNAs have been associated with PD and could be potential therapeutic targets, as miRNAs are able to specifi-cally regulate the expression of known PD genes and gene products [12]. Among them, miR-133b targets pituitary homeobox 3 (Pitx3), being involved in DNa neurons differentiation and activity, miR-7 as a well know target of a-synuclein with important role on oxidative stress medicated cell death and let-7 and miR-184 with catalytic E2F1 and DP activity against neurons survival [28],[29]. Similarly, five downregulated genes were identified as key molecular markers, among them MAPK8 and SDHC and three miRNAs (miR-126-5p, miR-19-3p and miR-29a-3p), in a meta-analysis study through bioinformatic analysis of multiple datasets obtained from Gene Expression Omnibus datasets [24]. Mitochondrial ATP synthesis-couples electron transport, branched-chain amino acid catabolic process, organelle envelope lumen and oxidoreductase activity acting on NAD(P)H can be highlighted among the main GO categories. Most potential biomarkers, including NDUFS1 (NADH: Ubiquinone oxidoreductase core subunit S1) and COX4I1 (Cytochrome C oxidase subunit 4I1), have been extracted while cross-platform datasets are described suggesting significant monitoring among prognostic predictors and novel therapeutics challenges related to PD [24]. Two microRNAs, miR-7 and miR-153, have been found to regulate endogenous a-synuclein levels and could be included as potential therapeutic strategies for modulating protein levels in PD, as miR-7 may inhibit protein expression and therefore to play a role against mediated cell death via oxidative stress [30]–[32]. Moreover, genetic polymorphisms of miRNA related sequences are highly associated with PD risk. FGF20 polymorphism in two 3'UTR single nucleotide polymorphism (SNPs) and one intronic SNP have been described as significant risk factors in a USA family study [33]. Furthermore, elevated levels of FGF20 led to upregulation of α-synuclein and predispose to PD [29]. A meta-analysis of published miRNA expression studies in brain, blood and CSF including idiopathic PD versus normal subjects and extracted data was performed to dissect differentially expressed miRNAs. miRBase was used for quality control and 160 meta-analyses resulted in 13 miRNAs with levels determined significantly modifying at least three independent studies. Among them, hsa-miR-221-3p, hsa-miR-214-3p and hsa-miR-29c-3p were found in blood and hsa-miR-132-3p, hsa-miR-497-5p and hsa-miR-133b in brain [34]. Moreover, plasma hsa-miR-671-5p, hsa-miR-19b-3p and hsa-miR-24-3p were reported differentially expressed in a study including PD patients, multiple system atrophy (MDA) patients and healthy controls using the 3D-Gene Human miRNA oligo chip and gene ontology process was performed using MetaCore [35]. Elevated levels of miR-27a were identified in the plasma of 25 patients diagnosed with PD while miR-142-3p and miR-222 were found downregulated as well as miR-30, miR-29, let-7, miR-485 and miR-26 [36],[37].

Genome wide association studies (GWAS) were performed to determine the association between sporadic PD with variety of genes with emphasis on SNCA encoding a-synuclein, leucine- rich repeat kinase 2 and MAPT appropriate of tau [38]. In a different study, integrative analysis of whole-blood gene expression for idiopathic PD patients was conducted and the identification of familial PD patients with the LRRK2 G2019S mutation was revealed among a panel of 113 candidate marker genes [39]. Lastly, it is widely known the integral purpose of microbiota in inflammation and immunity and the active role which implicates in PD. On this basis, analysis of microbiota in the blood of PD patients compared with healthy individuals using 16S ribosomal RNA gene sequencing determining a total of 29 taxa with distinct multitude between healthy and PD groups while higher magnitude of bacteria was determined in the PD patients [40]. Moreover, confirmation between the microbiota and PD clinical parameters was carried out, revealing specific taxa passively associated with disorder duration. Taking out certain limitations, including the widespread population or the huge pool of samples, association between taxonomic and non-motor symptoms (NMS) showed negatively correlations for *Helicobacter* and *Mucispirillum* with NMS scopes while no genus in blood was referred to Mini Mental State Examination and Montreal Cognitive Assessment scores. In the light the observation of significant alterations of the microbial 16S rRNA gene in PD patients, this study clearly links the fundamental role of the microbial motif to the pathogenesis of PD [40]. A transcriptome-wide association study (TWAS) approach was designed to prioritize candidate PD genes and to better elucidate the primary key molecules that underlie PD genetic risk players. Using large-scale transcriptomic datasets, sixty-six genes were prioritize whose predicted expression or splicing levels in peripheral monocytes cells and in dorsolateral prefrontal cortex are significantly associated with PD manifestation [41]. This study comes as a continuation of a 2017 GWAS which highlighted over 41 genetic susceptibility loci and their association with late-onset PD [42]. According to these evidences, any connection between PD susceptibility and *LRRK2* arise unlike to previous studies [43]. According to Sekiyama et al. it is expected that the knowledge gained from these genomic analyses will be applied to early diagnosis based on genetic testing in the future; however, GWAS have some limitations which should be overcome such as the heterogeneity of SNPs across the patients and healthy controls, possible variants in diseases and epigenetic modifications [44].

## 4. Bioinformatics-driven search for network and pathway analyses

**Figure 2.**
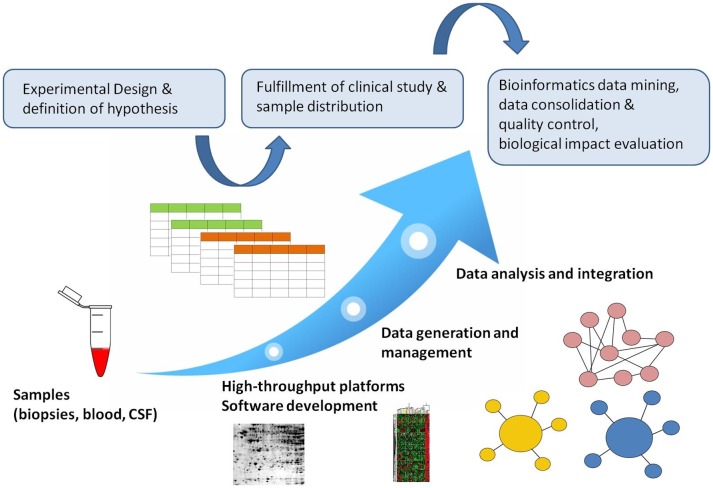
Analysis workflow including bioinformatics pipeline and data integration system.

Bioinformatics deals with the development and validation of computational methodologies for the analysis, clustering and validation of molecular and clinical indications, with the aim of diagnostic predictors identification and treatment. The availability of high-throughput technologies indented for clinical applications substantially helps to establish bioinformatic platforms for data pre-processing that could be applied to the storage and integration of homogenous data, the extraction of updated clinical knowledge with analysis pipelines as well as developing therapeutic approaches to consulting patients when interpreting results [45]. According to these pathways, differentially expressed genes, proteins and metabolites can be determined in combination with the identification of dysregulated networks associated with the candidate biomarker (Figure 2).

Many studies present extensive bioinformatics analysis either at gene level or miRNA expression to reveal important molecular markers associated with potential therapeutics for PD. In a recent study by Dong et al., the identification of global miRNA and mRNA expression profiles of normal individuals and patients with PD was carried out using Gene Expression Omnibus database [46]. The levels of 88 differentially expressed miRNAs were observed using well know processes like starBase database while the Cytoscape software was utilize to execute the miRNA-gene network associated with PD evidences. Among the potent expression profile datasets miR-590 and its target SPRY2, miR-142-3p linked to methylenetetrahydrofolate dehydrogenase and miR-338 associated with the expression of cytochrome *c* oxidase IV were found differentiated while GO analysis indicated that synapse and dopamine metabolic process along with biogenic amine biosynthetic pathways were implicated [46].

A web portal which can visualize mouse transcriptomic data associated with neurological disorders has been designed, supporting also selection of the ideal mouse model for follow-up investigation. It summarizes data into a centralized pool including annotations to each study for comprehensive exploration [47]. An innovative bioinformatic process called “Expression Data Up-Stream Analysis” (EDUSA) was developed for the necessity of analysis and categorization of genomic events to obtain a coherent explanation of the mechanism of the disease, showing the different stages of disease development by collecting a single-point data [48]. A brief overview of disease-oriented genomic expression profiling data analysis using EDUSA begins with sample collection, the analysis of differentially expressed genes, the categorization of over-represented biological families using Expression Analysis Systematic Explorer (EASE) software, the elimination of similar groups applied to conjugated gene clusters and is completed by inter-process interaction as well as upstream and downstream analysis and hierarchy identification. Among the advantages of this highly promising process is the rapid identification of genes and pathways affected by disorder through tissue samples obtain from both patient and healthy groups. GEO2R software was used to highlight disease-affected genes and EASE software for disease-pertinent approaches [48]. Furthermore, the upstream versus downstream hierarchy of the method was performed and the current misfolded protein theory associated with PD molecular pathways was confirmed by the extracted results. According to this process, it was suggested that the pathway of ribonucleic acid metabolism is involved in PD, which is particularly demonstrated in neurodegenerative disorders. In addition, malfunction of the transport system could be implicated in the initial phase of neurodegeneration unlike the mitochondrial dysfunction that occurred at a later stage.

In a recent study, a software called TRAM (Transcriptome Mapper) was used and meta-analyses was conducted on brain PD and healthy controls microarray data to ascertain DA neuronal transcription alterations by combining multiple data sets from independent studies [49]. This approach indicated the deregulation of specific genomic regions and loci implicated in functional pathways related to neurodegeneration along with genes and non-coding RNA transcripts not yet associated with the disorder such as GPNMB gene which encodes a transmembrane protein and was found overexpressed in substantia nigra of PD samples as well as *NPTX2*, *DEFA3* and *DEFA1*. In a recent review by Glaab, it is proposed that modifications of known cellular pathways and molecular processes unraveling could explain systems-level variations in omics datasets. To answer this question, distinct pathways and methodologies can be extracted from well-known databases such as Kyoto Encyclopedia of Genes and Genomes, Gene Ontology database, BioCarta and Reactome [50]. In this work, the author proposes to categorize omics-based pathways and geneset enrichment into distinct four families. Specifically, over-representation analysis (ORA) corresponds to abundance data found in an omics dataset, whereas geneset enrichment analysis (GSEA) helps eliminate the need to describe a significant threshold. The last two are the Network Module-based Pathway Analysis (NMPA) and the Network Topology-based Pathway Analysis (NTPA). The former uses algorithms to investigate prior knowledge of intracellular networks, while NTPA approach exploits molecular network profiles to establish robust and pathway association scores. For each of these four pathway analysis groups, a variety of software tools is available, providing meaningful and visible results. These include the DAVID and GOToolBox over-representation analysis tools and the GSEA, GLOBALTEST and PADOG geneset enrichment analysis tools, as well as the FunMOD for network module-based pathway analysis and PWEA, PathNet and ToPASeq which are oriented towards the network topology-based pathway analysis. It should be noted here that the selection of the appropriate pathway process is strongly associated with prior computation of altered expression for the individual molecular marker in each omics set, while for pathway analysis of genome wide association studies and sequencing results biases linked to the connection disequilibrium and geneset amount must be reported. Trying to determine PD-associated molecular network changed, specific software tools are presented for either network perturbation analyses or causal reasoning analyses. These approaches are able to reveal the regulatory network involved in a related biological state, regulatory relationships among genes or protein cascades. BioNet/HEINZ, ClustEx and GenePEN are used for network disturbances analyses while Whistle, CausaIr and SigNet can be utilized for causal reasoning analysis. Moreover, machine learning prediction models and high-dimensional data are really important in this direction for categorizing of diagnostic samples and clustering for diseased sub-group layering [51]. A number of essential software tools for machine learning analyses of omics data for clustering categorization and visualization is also mentioned. mixOmicsm MLSeq abd ArrayMining may be used for multi-purpose machine-learning analysis, Limma and RankProd focuses on feature ranking and selection while GGobi and PlotViz are oriented to low-dimensional data display.

Lastly, immunological system and neurodevelopment were enriched in PD-related genes gene set (PDgset) in a study by Hu et al., including 242 genes associated with PD through assimilating data from GO, pathway and pathway crosstalk process. WebGestalt and ToppGene were used to examine the functional characteristics of the PD-related genes [52]. Undoubtedly, these network models not only elucidate the mapping of the global landscape of molecular interactions and regulations in PD but may also determine detailed networks and potential key molecules with regulatory role in PD for further experimental investigation.

## 5. Bioinformatics analysis and therapeutic strategies in PD

PD treatment begins at the onset of the disease, with more than 50% of dopaminergic neurons destroyed. An ideal therapeutic protocol would have already targeted the pre-clinical phase for reserving neurodegenerative effects [53]. Prosavin, a promising therapeutic approach that targets three key enzymes necessary for dopamine synthesis (tyrosine hydroxylase, GTP cyclohydrolase and aromatic acid decarboxylase), has reached a phase I//II clinical trial, however it could not alleviate progressive neurodegeneration [54]. Computational bioinformatics analysis of gene expression was utilized to determine potent molecular markers of the disorder. Two groups of PD patients and non-PD-controls were included and 1004 differentially coexpressed genes were observed, revealing upon network building and impact factor process characteristic transcription factors like HLF, STAT4, E2F1, EGF3, and TAL1 as potent key players of the initial point [55]. Among them, the first three molecules were indentified elevated in PD patients. It should be highlighted that gene expression profile of PD derived from Gene Expression Omnibus, postmortem human brains were received and the average delay between PD and control was 26.2 and 19.8 hours, respectively. While metabolic profiling provides an important insight into AD mechanisms, the findings may be conflicting and inconsistent between studies. Data mining and bioinformatics were also utilizing in an attempt to unravel novel therapeutics drugs for PD. Dopamine metabolism and cholinergic metabolic are mainly used as traditional pharmacological targets of anti-Parkinson's disease. According to this study, the ideal drug for either preventing or treating PD may be metformin hydrochloride or melbine. 250 differential genes were discovered during data analysis of PD brain tissues while 31 distinct “key” genes were proposed during gene enrichment and protein interaction processes [56]. It should be noted here that metabolic pathways, carbon metabolisms and methionine metabolism were determined through KEGG pathway analysis providing new potential PD treatment strategies. On the contrary, gene clusters that encode significant risk factors, such as members of the PARK family, can be identified together with PARK1/4 which maintains a synaptic vesicles supply PARK11 associated with cooperation with GBR10 and insulin receptors signaling and PARK19 which is important for regulating the molecular chaperone activity [14].

Neuroprotection focuses on the inhibition of primary neurodegenerative events, providing significant improvements in many PD patients, despite the clinical, etiological, and genetic heterogeneity of the disease. Given that there is an initial loss of phenotype for midbrain dopamine neurons in Parkinson's disease (PD) rather than neuronal death, neurorestoration could also be a promising category of potential treatment presenting placement of new cells including fetal nigral neurons into the striatum of patient [57],[58]. In these two directions, specific brain-derived neuroptrophic factors, muscarinic acetylcholine receptor antagonists and inhibitors of monoamine oxidase (MAO)-B such as rasagiline have been developed [59],[60]. Rasagiline is a second-generation type-B monoamine oxidase inhibitor for the treatment of patients with idiopathic PD and motor fluctuation through blocking dopamine metabolism [61],[62]. Two other MAO-B inhibitors are selegiline and safinamide; the first such as rasagiline acts as an irreversible inhibitor though a covalent bond formation with the active site of MAO-B as well a voltage- sensitive sodium channels blocker and glutamate release. The mechanism of safinamide comprises reversible selective MAO-B inhibition and modulation of glutamate release [63],[64]. Others strategies for PD treatment included inhibitors of dopamine-metabolizing enzymes such as catechol-O-methyl transferase (COMT) with or without MAO-B [65]. As the first PD treatment strategy was based on the use of dopamine precursors, such as levodopa (L-dopa) which is able to cross the blood–brain barrier (BBB), combination therapies were applied to reduce symptoms. Rasagiline in combination with levodopa has been used to improve the efficacy of treatment in patients with PD without significant events compared with levodopa monotherapy [66]. In another study, rasagiline and safinamide were studied in an attempt to minimize the effects without reducing the equivalent dose of levodopa [67]. However, safinamide reduces the annual mean equivalent dose of levodopa and may be associated with a reduction in dose-dependent side effects in the long term. New molecular pathways can be explored through high-throughput approaches and bioinformatics analysis and new genes can be identified which may be considered as potential targets for drug development for Parkinson cases.

## 6. Conclusions

The topological complexity of neuropathological vulnerability and transcriptional regulation in PD suggests the need to characterize in more detail the molecular mechanisms underlying disease susceptibility and progression. Gene and pathway analyses provide an effective tool for cross-disease connections and for the molecular effects of factors involved in disease risk. It is increasingly important to discover novel biomarkers at the onset of PD along with the necessity to establish new therapies to prevent increased neurodegeneration and disease progression. The availability of high-throughput technologies intended for clinical applications in combination with computational systems biology approaches and bioinformatics analyses support this valuable purpose.
